# 

**Published:** 1895-03

**Authors:** 


### CONTENTS.

ORIGINAL ARTICLES:

Two Successful Cataract Operations on a Dog. By ROBERT L. RANDOLPH, M.D. . . . 133

The Anatomy of the Large American Fluke (Fasciola Magna) and a Comparison with Other Species of the Genus Fasciola, S.ST. By CHAS. WARDELL STILES, PH.D. . . . 139

Hyovertebrotomy. By WILLIAM DOUGHERTY, V.S. . . . 147

Hydrophobia. By J. H. ADAMSON, D.V.S. . . . 148

Croupous Pneumonia in the Horse. By ARTHUR SALINGER, V.M.D. . . . 155

Coal-tar and Some of Its Medicinal Products. By W. L. WEST, SR. . . . 162

Stringhalt. By S. J. J. HARGER, V.M.D. . . . 165

The Action of Rattlesnake Venom upon the Bactericidal Power of the Blood-serum. By CHARLES B. EWING, M.D. . . . 170

Some Interesting Post-mortems. By A. G. ALVERSON, V.S. . . . 176

SELECTIONS:

Legislation—Pennsylvania . . . 179

REVIEW OF VETERINARY SANITARY MEASURES . . . 179

EDITORIALS:

Missing Journals—Embargoes Against American Beef Products . . . 181

The Closing Days of Our College Courses . . . 182

EDUCATIONAL . . . 183

SOCIETY PROCEEDINGS:

Massachusetts State Veterinary Medical Association . . . 184

The Montreal Veterinary Medical Association . . . 185

Pennsylvania State Veterinary Medical Association . . . 189

A Pennsylvania Alumni Association of the American Veterinary College . . . 191

Western Ontario Veterinary Association . . . 192

Annual Meeting of the Oneida County Veterinary Medical Society . . . 193

Keystone Veterinary Medical Association . . . 194

Veterinary Medical Association, American Veterinary College . . . 194

McGill University . . . 195

PROGRESS IN THE ART OF FARRIERY . . . 196

NEW INVENTIONS—ITEMS OF INTEREST . . . 197

